# Friends with benefits: The effects of vegetative shading on plant survival in a green roof environment

**DOI:** 10.1371/journal.pone.0225078

**Published:** 2019-11-14

**Authors:** Axton C. Aguiar, Sharon A. Robinson, Kristine French

**Affiliations:** Centre for Sustainable Ecosystem Solutions, School of Earth, Atmosphere and Life Sciences, University of Wollongong, Wollongong, NSW, Australia; Georg-August-Universitat Gottingen, GERMANY

## Abstract

Green roofs help ameliorate some of the adverse social, economic and environmental effects of urbanisation. However, green roofs are harsh environments for plants, as they must cope with shallow soils, low nutrient availability, high solar radiation, low water availability and high pollution/disturbances. The effect of shade plants on vegetation survivability was investigated using green roof mesocosms with four different native species pairs (shade plant and a target ground cover plant). To examine the effect of shading and competition on plant growth and survival, plant pairs were subjected to four treatments; naturally shaded with a shade plant shading the target plant, artificially shaded with an artificial plant shading the target plant, unshaded natural which had a trimmed shade plant providing no shade to the target plant and an unshaded treatment with the target plant being the sole occupant of the mesocosm. The experiment ran for 11 months with measurements taken monthly to record growth and visual health of the target plant. Soil moisture and biomass data was collected at the end of the experiment. Overall, natural shade treated plants had the highest biomass while unshaded plants had the lowest biomass. Contrary to our predictions, the shaded artificial and the unshaded natural had similar moderate biomass. This similarity suggests that while shading had a positive influence on plant growth, there was also a positive influence of growing with a shade plant which is not accounted for by shading. The results highlight the complexity of biotic relations between plants and emphasises that the presence of a nurse plants can be benefit to the survival and growth of other species within a green roof ecosystem.

## Introduction

Urban areas have been expanding at an increasing rate [1,2]. Currently, 4 billion people inhabit urban areas with this projected to grow to 10 billion by 2070 [1]. The consequence of this growth has been a host of negative social, environmental and economic impacts [3,4] with increasing pressure assigned to urban green spaces to help ameliorate these impacts. Not only do green spaces provide essential ecosystem services, but they also improve and contribute to human wellbeing [5–7].

Intensive green roofs have been actively promoted to provide various benefits such as counteracting or controlling the ‘urban heat island’ effect [6,8], as refuges for biodiversity in urban environments [9,10] increasing productivity in work spaces [11] and improving health and wellbeing. There have been challenges that have hindered the widespread implementation of intensive green roof such as the increased costs associated with a green roof, increased water consumption, fertilizer runoff and the reduced aesthetics due to the loss of vegetation. Most of these drawbacks can be overcome with careful plant selection.

Species selection is one of the critical components of a green roof, as it dictates not only the success but also the benefits [12,13]. However, selecting plants for a green roof poses its own set of challenges. Plants have to contend with resource shortages, shallow growing substrates, increased pollution and high abiotic stressors. This restricts the types of plants that can be used on a roof, and represents a gap in the research. There have been different methods of selecting species on a green roof such as the habitat-template approach proposed by Lundholm (2018)[14]. The approach is based on the concept that novel artificial ecosystems can be modified to exhibit conditions similar to those presented by the vegetation’s natural habitat, which reduces the novelty of a roof environment for the vegetation [15]. Another approach is to use trait specific selection [16–18] which considers particular plant traits such as plant size, flowering rate, pollinators and aesthetic value [19]. The key assumptions of these models are that abiotic factors are the critical drivers limiting plant growth and survival on a green roof. However, there is a growing body of evidence highlighting the importance of biological interactions in shaping and influencing survival and growth of vegetation[20,21].

Species composition of vegetation communities is driven by their interactions with biotic and abiotic factors. Historically, abiotic factors (soil moisture, temperature, nutrients and pH) were thought to have a more significant influence on species establishment and survival [22], while biotic interactions, such as competition and facilitation, were considered to have a more significant role in species co-existence and structure [23,24]. The presence or absence of these factors along a gradient can be a factor in influencing the variation both in, and between, vegetation communities [25]. These variations can occur from small spatial scales up to global scales.

While initially negative species interactions and physical environments were considered the dominant drivers of species distribution and abundance [26], there is now a consensus that facilitation, plays an equally important role in determining community structure [24]. However, competition and facilitation do not occur in isolation with each other. For example, nurse plants aid in the establishment and survival of surrounding plants by ameliorating the microclimate and altering the soil properties (nutrients and water content)[27]. Nurse plants in alpine conditions provide shade and shelter for seeds allowing them to germinate and grow. As the seedlings mature, they compete for resources with the nurse plant, shifting the relationship from commensalism/facilitation to competition [28]. The interaction of facilitation and competition also varies with life history. It has been demonstrated experimentally that facilitation provided by neighbouring plants facilitated the establishment and growth of younger pines in severe environmental conditions[29]. However, this relationship was reversed as the pines matured exhibiting higher levels of competition.

Nurse plants facilitate other plants within their canopy by modifying the microclimate and soil [28,30]. The canopy ameliorates abiotic weather conditions by trapping heat in colder alpine conditions [30] or providing shade in hotter conditions [31], increasing soil nutrients by increasing decomposing matter from leaf litter [32], reducing wind [33] and increasing soil moisture content [34]. Nurse plants increase species richness [35], abundance [36] and play an essential role in improving the survivability of species [36,37].

Using nurse plants in urban areas is not a new concept. Horticulturally nurse plants or “buddy planting” have been used for decades to improve plant survivability, fruiting success, and plant growth [38]. However, at present, there is limited information on how nurse planting may improve growth on green roofs and what influence the competitive effects may have. An example of this is a study conducted by Butler and Orias [31] investigated the use of *Sedum album* (Crassulaceae) as a nurse plant to facilitate the growth of *Agastache rupestris* (Lamiaceae) and *Asclepias verticillata* (Asclepiadaceae). They concluded that *S*. *album* might have acted as a competitor during periods of high-water abundance and that after three months it had a negative impact on the biomass of *Agastache rupestris* and *Asclepias verticillata*.

We investigated the effects of shade plants on plant growth and survival in a green roof environment. We refer to these species as shade plants as we considered that their advantage would primarily be in shading target plants. We aimed to examine the effects of shade and competition on four different plant species in a simulated green roof environment. We predicted that if the growth of rooftop vegetation is most affected by high irradiance and heat exposure, then protecting vegetation with shade will increase growth. However, if the growth of rooftop vegetation is more strongly influenced by competition, then exposing species to a shade plant will decrease growth.

## Methods

### Study site and green roof array

The study took place at the ecological research centre (ERC) at the University of Wollongong, Australia (34°25′59″S 150°52′59″E). Wollongong has an oceanic climate with humid subtropical influences. Rainfall is associated with the orographic lift caused by the escarpment; on average Wollongong, experiences 1300 mm of rain a year with the wettest months being February and March. Green roof mesocosms were constructed in December 2015 on top of a large concrete slab simulating the roof of a building. All mesocosms were North-South orientated on the slab to ensure that each setup received a comparable amount of exposure to solar radiation.

**Fig 1:**
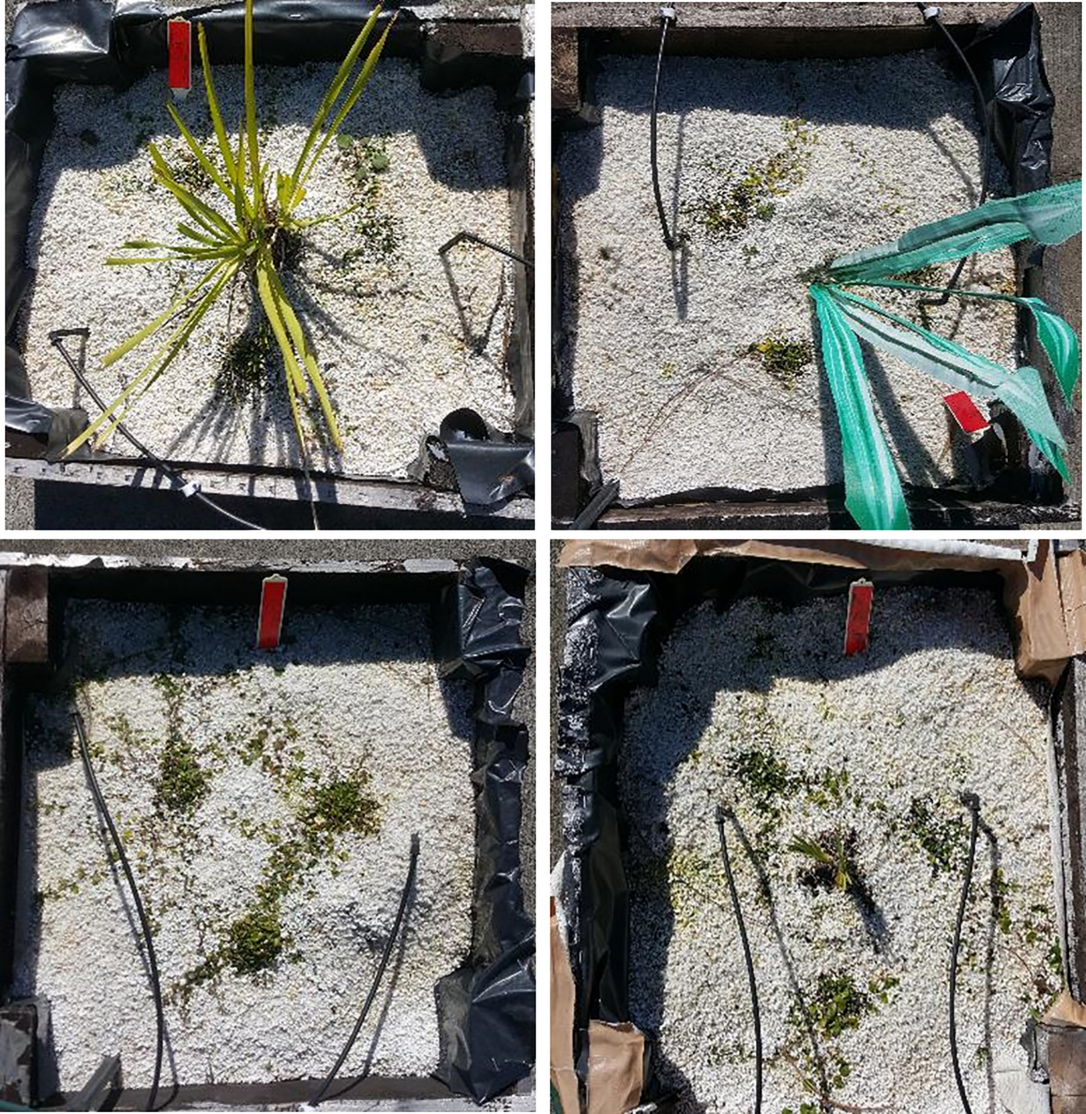
Pictorial representation of the 4 treatments used in the experiment. Treatments: from the top left in a clockwise direction, shaded natural, shaded artificial, unshaded natural, unshaded (example of *Lomandra longifolia* and *Dichondra repens* pair).

Each mesocosm was constructed using technical guidelines published by the city of Sydney [39] in an attempt to replicate green roof construction. The frame of the green roof was built using a lightweight timber, which was constructed into a rectangular box (0.5*0.6*0.3 m) (Fig 1). The green roofs were created using a layered design: -

- Vegetation layer—the species used in the experiment were native sandstone species (forbs, low shrubs, succulents and grasses) found along the coast near Sydney.
- Growing layer– 20 cm deep consisting of a mix of organic and inorganic components. We used perlite (2–3 mm), vermiculite (2 mm) and topsoil (1–4 mm) in a ratio of 4:2:1. The organic matter was kept low (<20%) to mimic the nutrient-poor sandstone soils native to the region. The soil had bulk density of ~210 kg/m3 with a water holding capacity of 72% v/v
- Filter membrane—this consists of a thin membrane that prevents the soil from falling into the drainage membrane. We used a broad-gauge shade cloth
- Drainage layer– 14 mm crushed terracotta
- Protection mat—a Thermoplastic sheet layer to provide root and chemical protection
- Waterproofing membrane- We used a liquid applied treatment of bitumen emulsion.

### Experimental design

Testing all the species pairs listed in the local green roof guides was outside the scope of this project due to space limitations. We therefore employed a more general approach where species were selected from native habitats that experienced harsh abiotic conditions with poor nutrient soil and high drought stress, such as foredunes of beaches and exposed sandstone habitats. Accounting for experimental space and replication needed we were able to test four pairs of plants, each comprising a shade plant and a target plant (Table 1). All plants were purchased from the same nursery and were matched for size. All target plant species grew along the ground and were no taller than 10 cm. We tested four groups (Table 1) that were subjected to four treatments (described below) with five replicates per treatment (20 mesocosms per group).

**Table 1:** Species groups used in the experiment, each group consists of one shade plant and one target plant.

Group	Shade Plant	Life form	Target plant	Life form
A	*Lomandra longifolia*	perennial herb	*Dichondra repens*	perennial herb
B	*Callistemon citrinus*	shrub	*Grevillea lanigera* (prostrate form)	Shrub
C	*Dianella caerulea*	perennial herb	*Carpobrotus glaucescens*	succulent perennial herb
D	*Correa alba*	shrub	*Viola hederacea*	perennial herb

Each pair was grown for 11 months, the mesocosms were set up with the shade plant in the middle surrounded by 3 ground cover plants positioned 5 cm away from the shade plant in a NE, NW and S orientation. The four different treatments (Fig 1) were:

- Shaded natural (SN)–consisted of a live shade plant shading a target plant. This treatment emphasises the interaction of both above ground shading as well as the belowground root interaction.
- Shaded artificial (SA)–consisted of an artificial plant shading a target plant. The artificial plant consisted of indoor artificial plants that replicated the shade provided by the living plant in the shaded natural treatment. This treatment isolated the effect of shading by excluding any below ground/root interactions. The upper section of the plastic shading plant was replaced midway through the experiment as the plastic degraded due to UV damage.
- Unshaded natural (UN)–which consisted of a trimmed live shade plant providing no shade to the target plant. This treatment is the opposite of the previous treatment where the below ground interaction is isolated by minimising any above ground shading effect.
- Unshaded (U)–with the target plant being the sole species of the mesocosm. In this control treatment the target plants had neither interspecific competition nor facilitation (shade).

The mesocosms were set up a month before the start of the experiment to allow plants to acclimatise. All plants were watered via a drip system twice a day at 7 am and 7 pm with 2.5 L over 15 minutes based on the standard watering regime for intensively managed roof gardens in the Sydney region (A. Aguiar unpub. data). A slow release fertiliser (20 g of Osmocote Exact Standard 3-4M; 7.1% NO3-N, 8.9% NH4-N, 9% P2O5, 12% K2O) was applied twice during the experiment on days 1 and 175, to ensure that each treatment received an equivalent amount of nutrients.

### Soil moisture content

To investigate how soil moisture was affected by shade plants, soil water was measured after 11 months using a 50 ml container with a diameter of 40 mm (gravimetric method described in [40]. A sample of soil was collected from each mesocosm to a depth of 5 cm from the south side of each mesocosm, making sure to stay 10 cm away from any plant. Soil samples were weighed then placed into a drying oven at 80°C for 48 hours before reweighing. The volumetric water content in the soil sample was determined by multiplying gravimetric water content by the soil bulk density.

### Soil surface temperature

To investigate if shade plants ameliorate soil surface temperatures, the temperature of all mesocosms was logged with a K-type thermocouple comnected to a data logger (Easylog, EL-USB-TC) at 13:00 and used to compare shade performance in full sun. The temperature probe was inserted below the top layer of the substrate (3–5 cm deep) on the southern side of the shade plant.

### Biomass growth analysis

At the end of the experiment, ground cover target plants were harvested and separated from shade plants. Roots of target plants proved easy to separate from shade plants due to the loose structure of the soil and were washed over a sieve to clean, care was taken to ensure that any fine roots were collected and kept with the sample. For both the growth and biomass measurements the data from 3 target plants in each mesocosm was summed, because in the case of *D*. *repens* and *V*. *hederacea* it was impossible to delineate individual plants in some treatments. Growth measurements were collected (described below) and above ground and below ground biomass weighed separately. Plant material was placed in a drying oven at 75°C; a subsample was weighed daily until constant weight was achieved (72h).

### Growth analysis

After the roots had been washed for biomass collection, three measures of growth were recorded: 1. length of shoot/runner (for plants with branching shoots/runners the branches were added to the total length), 2. number of shoots/runners and 3. number of leaves. The number of leaves was not recorded for *G*. *lanigera*, due to the high number of leaves per unit area found all along the stem, instead stem length was seen as a better measure of growth.

### Statistical analysis

Each species was analysed separately. Root and shoot biomass, growth parameters (number of leaves, shoots and shoot length) were compared amongst treatments using single factor ANOVA after testing for normality (Shapiro-Wilks) and homogeneity of variance (Little et al., 2006). Significant interactions were further investigated using multiple comparison tests (Tukey's HSD). Soil moisture was tested using a 2 factor ANOVA.

For the soil temperature analysis, due to the confounding effect of variables such as wind and rain, we decided to use a local weather station to select days which had no rain on the day before measurement, wind speed was below 10 km/h, and cloud cover was below 20%. After filtering days with the above criteria, we then categorised the remaining days into 3 temperature groups (High >26°C, Medium 16–26°C and Low <16°C). We only used soil temperature collected on these days for our analysis. Data was analysed by ANOVA with replicate mesocosms nested within experimental treatments (shaded unshaded).

## Results

### Environmental data

#### Soil moisture content

**Fig 2:**
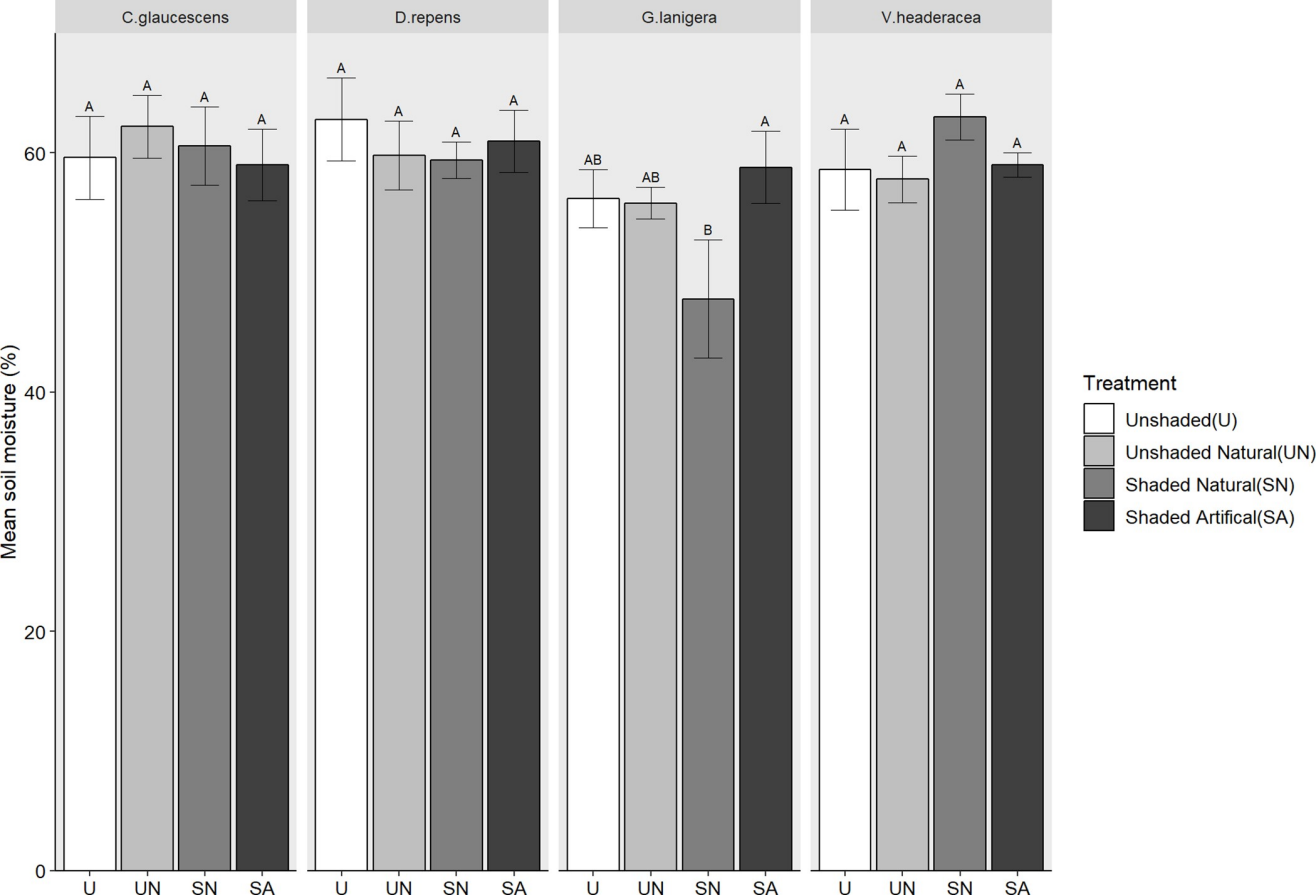
The effect of shading treatment on soil moisture content. Mean soil moisture content, across four different treatments U = Unshaded, UN = Unshaded natural SN = Shaded natural and SA = Shaded artificial. Letters denote statistical difference. Error bars are +SE, n = 5.

Only *G*. *lanigera* had any statistically significant difference between the treatments for mean soil moisture (*Fig 2*, Table 2). The significance was driven by the SN treatment having half the soil moisture content when compared to the other treatments; all other treatments had statistically similar soil moisture content.

**Table 2:** Summary statistics from analysis of variance carried out comparing the effect of shade plants on biomass, growth and soil moisture in a green roof environment.

Species	Test	F	df	SS	P
*C*. *glaucescens*	Biomass Above	20.239	3,16	224.822	<0.0001 *
	Biomass Below	4.2114	3,16	21.961	0.0224*
	# of leaves	8.9804	3,16	91.6	0.0010*
	# of shoots	12.296	3,16	5.53	0.0002*
	Shoot Length	44.5	3,16	717.50	<0.0001 *
	Soil moisture	0.2035	3,16	9.78	0.8925
*D*. *repens*	Biomass Above	36.536	3,16	24.57	<0.0001 *
	Biomass Below	40.388	3,16	257.54	<0.0001 *
	# of leaves	51.019	3,16	4832.8	2.057e-08
	# of shoots	53.46	3,16	220.55	<0.0001 *
	Shoot Length	194.27	3,16	16286	<0.0001 *
	Soil moisture	0.318	3,16	11.65	0.8117
*G*. *lanigera*	Biomass Above	29.33	3,16	7.56	0.0001*
	Biomass Below	6.916	3,16	22.82	0.001*
	# of leaves	19.039	3,16	268.32	<0.0001 *
	# of shoots	7.266	3,16	7.266	0.0027*
	Shoot Length	###	###	###	###
	Soil moisture	3.51	3,16	270	0.0396*
*V*. *hederacea*	Biomass Above	62.678	3,16	8.2556	4.605e-09*
	Biomass Below	31.707	3,16	158.77	5.759e-07*
	# of leaves	31.411	3,16	3126	6.13e-07
	# of shoots	15.76	3,16	241.2	4.901e-5*
	Shoot Length	71	3,16	10031	1.807e-9*
	Soil moisture	4.4588	3,16	358.15	0.1855

*denotes statistical significance

### indicates no data collected

#### Soil temperature

**Fig 3:**
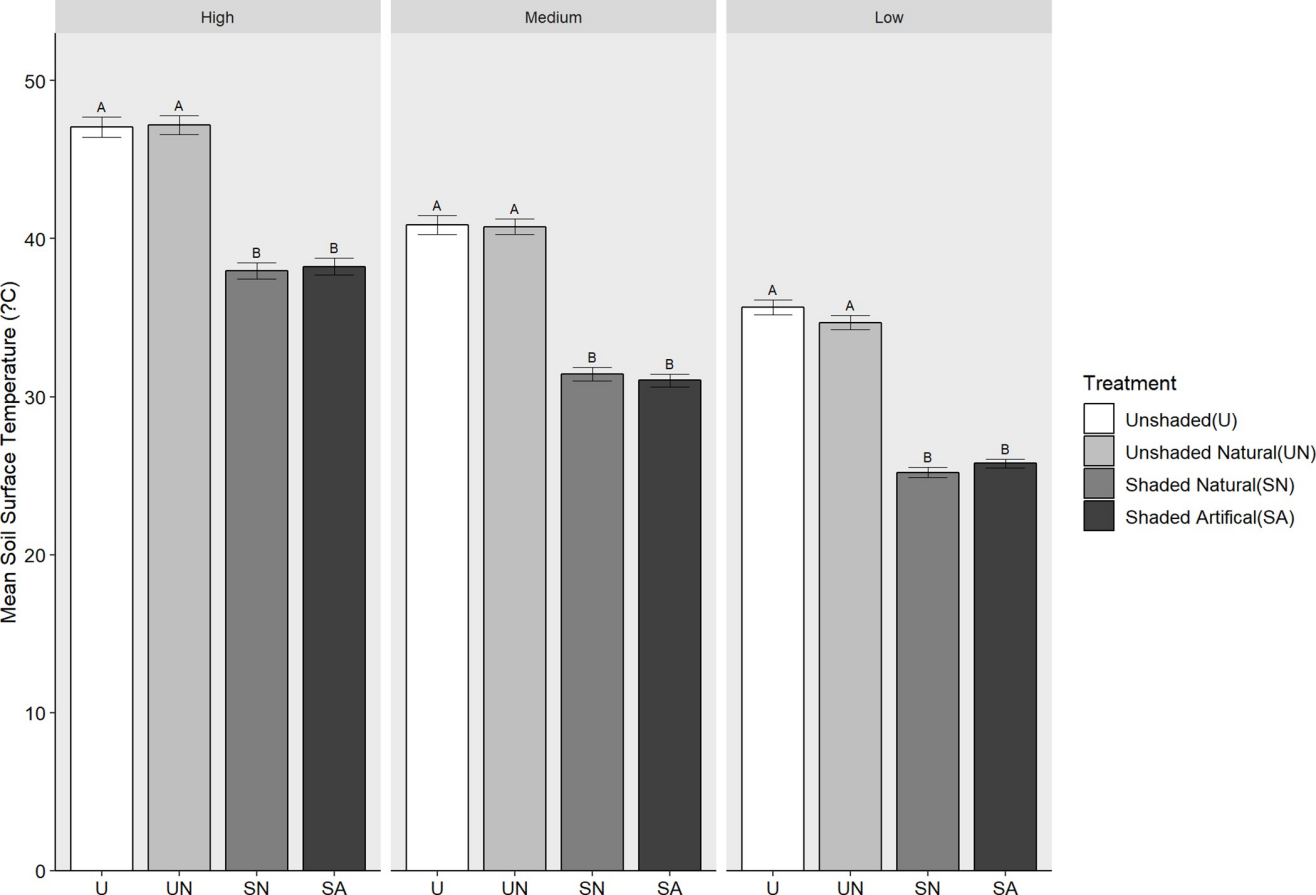
Effect of shading on soil temperature. Difference between midday soil surface temperature across four treatments; U = Unshaded, UN = Unshaded natural SN = Shaded natural and SA = Shaded artificial on days with high (>25°C) medium (16–25°C) and low (<16°C) ambient temperatures. Error bars are +SE, n = 20.

Shading had a significant effect in reducing soil temperatures when comparing midday soil temperatures on high medium and low days throughout the year (High, F_3,304_ = 124.16,P = <0.001; Medium,F_3,304_ = 171.84,P = <0.001; Low F_3,304_ = 207.51,P = <0.001)(*Fig 3*).On average there was a 15°C difference between shaded and unshaded treatments. This effect varied with ambient temperature. On hot days the difference between shaded and unshaded was close to 25°C.

### Biomass

**Fig 4:**
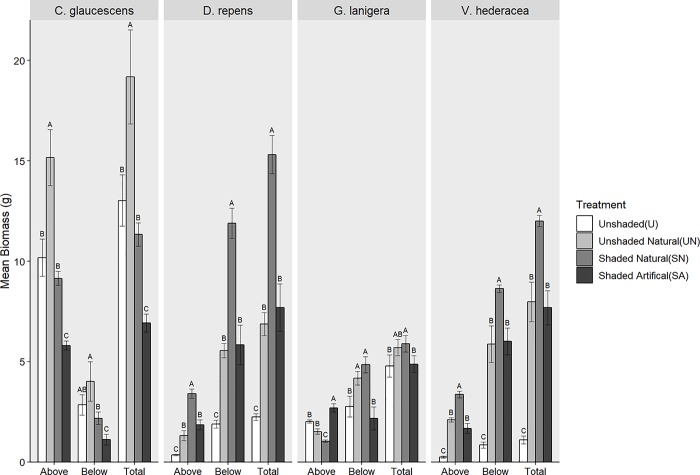
The effect of shading on plant biomass. Comparison of the effect of four different shade treatments on mean pooled dry biomass. Above ground, below ground and total biomass for each species were analysed separately, and letters denote statistical difference within factors (above and below). Error bars are +SE, n = 5.

All treatments, except for *C*. *glaucescens*, exhibited an increase in both above and below ground biomass when compared to the control (*Fig 4*, Table 2). For *D*. *repens* and *V*. *hederacea* shade increased biomass by at least a factor of 2 (*Fig 4*). The SN treatment had the highest increase in biomass with the UN and SA treatments having statistically similar increases in biomass (*Fig 4*, Table 2). There was thus no evidence of competition, instead, both treatments with a shade plant exhibited an increase in biomass when compared to U and SA treatments (*Fig 4*).

The results were more complex for *C*. *glaucescens* and *G*. *lanigera*. For *C*. *glaucescens* there was a greater increase in above ground biomass when compared to the below-ground biomass (*Fig 4*, Table 2). For above ground biomass, the shade treatments (SA and SN) exhibited lower or similar biomass to the control treatment (*Fig 4*, Table 2). For *G*. *lanigera*, shade did have a positive effect on growth. However this was only seen for above ground biomass in the treatment with the artificial plant (*Fig 4*). In contrast, both treatments with the shade plant exhibited higher below ground biomass at the expense of above ground biomass (*Fig 4*). This effect was strongest in the SN treatment which had the lowest above ground biomass and the highest belowground biomass (*Fig 4*, Table 2).

### Growth

**Fig 5:**
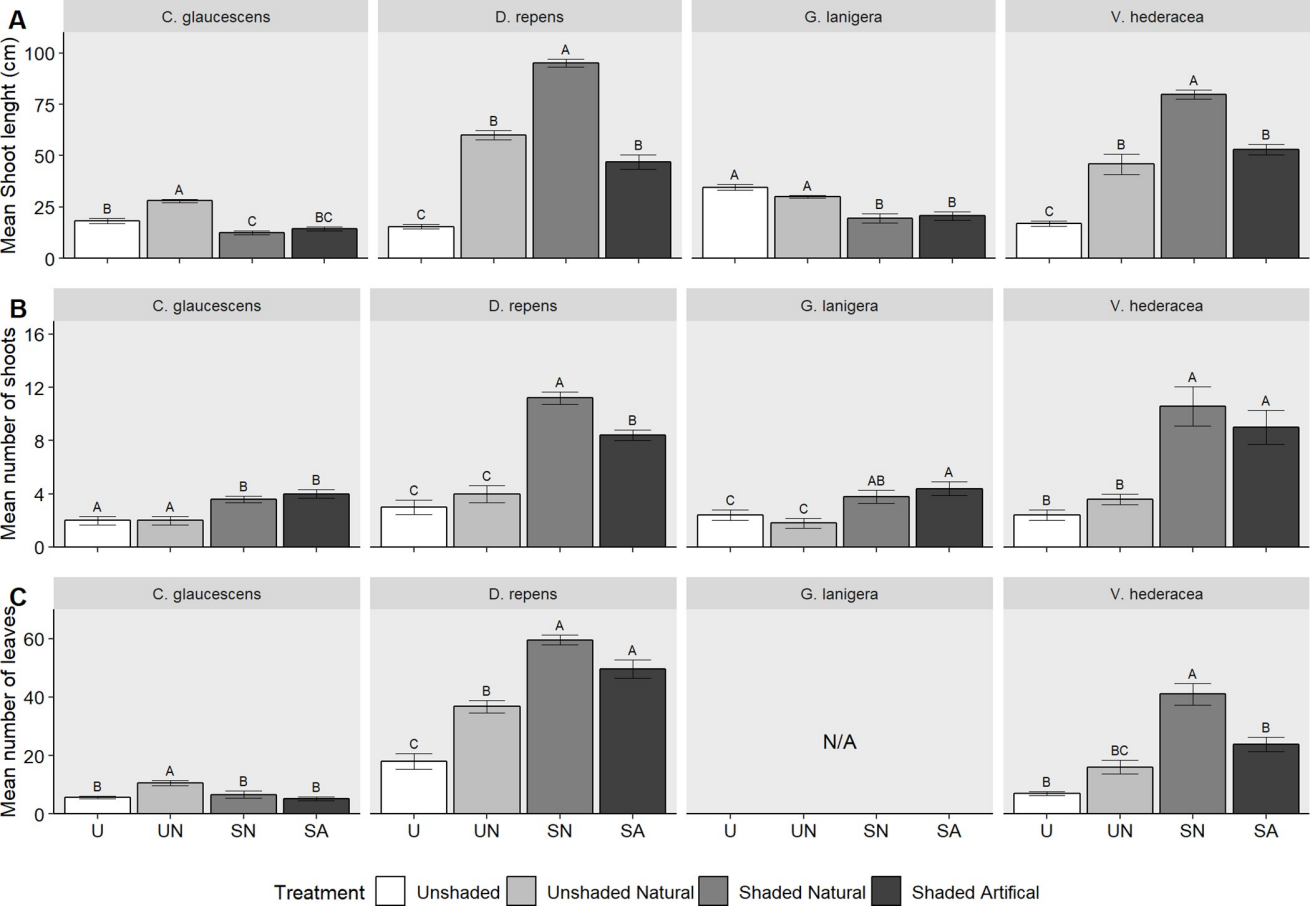
The effect of shading on plant growth. Comparison of the effect of four different shade treatments on the mean number of shoots (a), number of leaves (b) and shoot length (c), for four different species. Error bars are +SE, n = 5. Separate ANOVAs and multiple comparisons were done for each species; different letters signify statistically different means.

While the overall trend of the growth data was similar to that of the biomass data, each species exhibited subtle differences to the different shade and competition treatments. There was a significant effect of shading on the number of shoots (*Fig 5A*, Table 2). All four species exhibited a lower number of shoots in the unshaded treatments (U and UN) when compared to the shaded treatments (SN and SA). For *V*. *hederacea* and *D*. *repens* shading doubled the number of shoots (*Fig 5A*).

Changes observed in the number of leaves (*Fig 5B*, Table 2) mirrored the biomass changes with shade treatments (SN and SA) having fewer leaves for *C*. *glaucescens* and more leaves for *D*. *repens* and *V*. *hederacea*. *C*. *glaucescens* exhibited the highest number of leaves in the unshaded treatment with a shade plant. For shoot length (*Fig 5C*) the positive effect of shade (SA) was equal to the positive impact of the shade plant (UN) for both *V*. *hederacea* and *D*. *repens*, with the combination of both in the SN treatment achieving the highest shoot length (*Fig 5C*, Table 2). *G*. *lanigera* had a significant effect on shoot length for the unshaded treatments having longer shoots when compared to the shaded treatments.

## Discussion

In this work, we assessed the performance of four different ground covers under green roof conditions. We focused on the effects of facilitation and the below-ground competition of a shade plant on the focal species. Overall, we identified that facilitation played a more significant role in influencing plant growth compared to competition. Below we discuss some of the mechanisms affecting plant growth.

### Shading

The main facilitative mechanism, shading, positively influenced plant growth although it differed amongst species. *V*. *hederacea* and *D*. *repens* exhibited increased biomass when subjected to shade in both natural and artificial treatments, suggesting that for these two species, shade plants facilitate growth through shading from continuous solar exposure. *G*. *lanigera* also exhibited increased biomass when subjected to artificial shade, however any positive benefit when exposed to shading from a shade plant was overwhelmed by the competitive response to the shade plant. *C*. *glaucescens*, on the other hand, had a negative reaction to shade.

Shading ameliorates excessive solar radiation and may lower soil temperatures or reduce water evaporation from soils. In our experiment, we found no reduction in water loss in shaded soils although soil moisture was reduced in *G*. *lanigera* due to competitive effects (see below). However, soil temperatures were affected and suggests that shade plants can ameliorate elevated soil temperatures, particularly on hot days. Soil temperatures were reduced by 15°C on hot days, but only by 8°C on days of moderate air temperature. This difference may be the main mechanism for facilitating root growth as high root temperatures are known to be lethal for the roots of plants [41].

Excessive solar irradiation is detrimental to plant growth. Green roofs are typically subjected to harsh environmental conditions [42], including excessive irradiation. A study conducted by Getter et al. [43]found sunlight to be one of the main abiotic constraints on a green roof. In his study, he found that full solar exposure reduced the growth of certain species of plants by nearly 90%. These results agree with our findings where the control treatment in our experiment had the lowest growth and biomass for three of our species (aside from *C*. *glaucescens*). High solar radiation leads to reduced photosynthetic activity due to the photoinhibition of PSII [44,45], retardation of plant growth [46], high leaf temperatures leading to stomatal closure and inactivation of enzymes leading to a decrease in growth [47]. Shading helps ameliorate some of the abiotic stresses on a green roof by providing a physical barrier that limits the amount of direct incoming solar irradiation. A gradient of shading is provided by a nurse plant depending on plant characteristics and the nearness of the target plant to the nurse plant creating areas of varying light intensities. Target plants will likely grow better if this approaches their optimum light range.

For species adapted to high light environments, neighbouring plants may cause deficient light intensities which could reduce the rate of photosynthesis. If light intensity drops below the compensation point, plant respiration will use up more photosynthates, including carbon, leading to reduced growth. The addition of shading in our experiment had a negative impact on the growth of *C*. *glaucescens*. *C*. *glaucescens* is typically found on exposed seaside cliffs and dunes where it must contend with full solar radiation, high salinity, high winds and low freshwater. It appears to be physiologically adapted to high solar exposure. One of the limitations of our experimental design is we did not have a measure of cover over time. Using a cover over time estimate we could have assessed if increasing cover by the nurse plant reduces cover of a target plant like C. *glaucescens*

If the shading of a shade plant is truly facilitative, then the growth habit of plants should reflect a desire to remain within the optimum light range, with a clustered growth habit. In the shade avoiding species, there is an increase in stem and petiole elongation to cover a wider area to receive more light [48]. This was observed in *C*. *glaucescens*, however, increases in stem number and length in *D*. *repens* and *V*. *hederacea* was accompanied by an increase in the number of leaves, associated with the overall increase in biomass in the shaded treatments and there was no evidence of shade avoiding behaviour.

### Competition

The lack of competition between some of the pairs of plants was unexpected as it has been well documented in other experiments that make use of nurse plants [49,50] and is ubiquitous in natural ecosystems. Some of the positive benefits of nurse plants are offset by the resource competition provided by adding another plant to the habitat [51]. However, in our experiment, only *G*. *lanigera* exhibited signs of competition with its shade plant, *C*. *citrinus*. Unlike the other species, in treatments with a shade plant (SN and UN), *G*. *lanigera* exhibited higher below ground biomass while above ground biomass was lowered. The trade-off effect observed in *G*. *lanigera* is a resource competition response whereby there is an increase in roots at the expense of shoots. We observed a reduction in available soil water when a shade plant was present (SN). [52] found that under light competition, plants allocated more resources to above ground growth. In our experiment, we observe that *G*. *lanigera* was allocating resources belowground to compete with the shade plant at the expense of its above ground biomass.

### Below ground facilitation

The other unexpected result was the facilitative effect that the shade plant had on the ground covers when they were not providing significant shade. By trimming the shade plant and limiting the shading benefit, we expected that plants would be under root competition without the confounding positive effect of shade. Interestingly, there was a positive below ground interaction that increased both above and below ground biomass of the shaded plant. In *D*. *repens* and *V*. *hederacea*, the facilitative effect of this below ground interaction was equal to the positive impact of shade in the artificially shaded treatment (SA). This benefit was also observed in the naturally shaded treatment (SN) where the positive benefit was often above that of the SA treatment.

One method of facilitation is the modification of the substrate through increased nutrients available to the plant due to leaf senescence and mycorrhizae [53]. In our experiment, all dead leaves were collected, and so contributions from leaf senescence are negligible. There is ample evidence in the literature demonstrating how mycorrhizae increase plant growth and survival [54] and ameliorate abiotic stressors [38]. Mycorrhizal networks may well transfer nutrients between the nurse plant and target plants through a common mycorrhizal network [55–57]. Xin -Hua He [55]investigated the movement of labelled nitrogen (N^15^) between nurse plant and surrounding plants and found nutrient exchange between the nurse plants and the target plants using a common mycorrhizal network. Similar evidence of below ground facilitation was found in vines ([58]).

## Conclusion

Our results highlighted the effectiveness of shade on a green roof in increasing growth for many species. While it was outside the scope of this experiment, future studies should investigate the effect substrate type has on the facilitative effect of a shading plant. For example, in this experiment we only used a substrate mix of perlite, vermiculite and soil which has a light colour. The facilitative effect of shading plants may be more pronounced in darker substrates as it may help keep substrate temperatures lower.

On the whole the use of shade plants is a practical option for improving the diversity of plant selection on a green roof. Not only do the shade plants enable species that are typically stressed by roof garden conditions, to be used, but they also contribute to the biodiversity and aesthetic benefits provided by the green roof. In our experiment, the species pairs were chosen at random with no optimising between shade plant and target plant. With careful selection of shade plants as well as proper planting densities aimed at improving abiotic conditions for a target plant, we might be able to increase the effect of the shade plant as well as reducing some of the competitive effects.

Green roofs can also be designed to maximise potential for plant-plant facilitation for example green roofs can be built with varying substrate depth to provide suitable habitat for shade plants while maintaining a lower overall weight across the green roof. Understanding the mechanisms of facilitation, particularly below ground, will be an essential action to maximise the success of green roofs. We can use shade plants to optimize the establishment and success of gardens in urban areas, to increase social and biodiversity benefits. While green roofs are growing in popularity care should be taken to carefully select and build green roofs to ensure long term success.
